# Fixed Drug Eruption Secondary to Four Anti-diabetic Medications: An Unusual Case of Polysensitivity

**DOI:** 10.7759/cureus.18599

**Published:** 2021-10-08

**Authors:** Dana Al Masri, Mohamad Fleifel, Kamal Hirbli

**Affiliations:** 1 Endocrinology, Diabetes, and Metabolism, Lebanese American University Medical Center, Beirut, LBN; 2 Internal Medicine, Lebanese American University Medical Center, Beirut, LBN

**Keywords:** fixed drug eruption, type 2 diabetes mellitus, anti-diabetics, polysensitivity reaction, magnesium stearate, excipient

## Abstract

We report a case of fixed drug eruption in a 58-year-old lady treated for diabetes with four pharmacologically different anti-diabetic agents that were used at separate times of therapy. Skin manifestations, including erythema, blisters, and ulcers, developed over the right leg each time after the initiation of metformin, gliclazide, vildagliptin, and empagliflozin; and disappeared following the discontinuation of the drugs. Magnesium stearate was the common excipient identified in the four agents. This is an extremely rare case of fixed drug eruption caused by structurally dissimilar drugs.

## Introduction

Cutaneous eruption is the most common adverse reaction attributed to a drug; however, fixed drug eruption (FDE) represents approximately 5-10% of general cutaneous reactions and 2.5-22% of cutaneous adverse drug reactions [1,2]. It is mainly characterized by skin lesions that recur at nearly identical anatomic sites upon repeated exposures to the same offending agent. The drugs most commonly implicated in FDE are analgesics and antibiotics. Although cross-sensitivity of two chemically related drugs has been previously described, FDE of two of more unrelated agents has rarely been reported. We report an FDE occurring secondary to the use of four unrelated anti-diabetic medications.

## Case presentation

**Figure 1:**
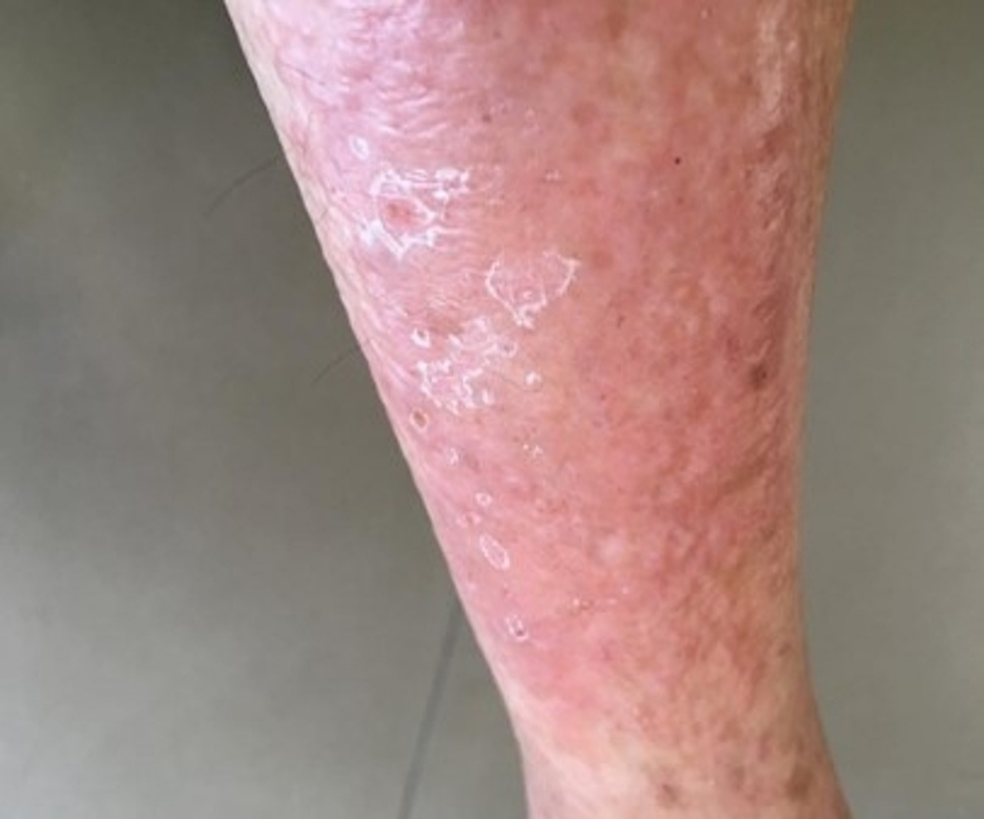
Dermatologic manifestations after metformin initiation

A 58-year-old lady, recently diagnosed with diabetes mellitus-type II in the past two weeks, presented to our clinic with chief complaints of itching and burning sensation over the right leg. It was accompanied by the appearance of blisters, ulcerations, and erythema (Figure 1). This was two weeks after the initiation of metformin. The patient did not exhibit any symptoms suggestive of cellulitis or erysipelas like fever, frank pain, or demarcation. There was nothing similar to any rheumatism associates or systemic autoimmune diseases; for example, dermatomyositis findings like heliotrope, shawl rash sign, Gottron’s rash, or any myopathy. The patient underwent a skin biopsy showing evidence of a lichenoid drug eruption. Despite it being an unusual causative agent, metformin was suspected as the drug causing this presentation because it was the only medication the patient started taking recently. Therefore, metformin was stopped and the rash disappeared a few weeks later.

The patient was then switched to gliclazide. She developed the same rash at the exact previously mentioned location. It also appeared two weeks after the initiation of this therapy. The described reaction vanished three weeks after gliclazide was discontinued.

After around two weeks of the introduction of vildagliptin, the same eruption appeared along the identical region. As described with the previous two agents, once the vildagliptin was withheld, the skin lesions subsided within a couple of weeks, with residual dark skin pigmentations.

**Figure 2:**
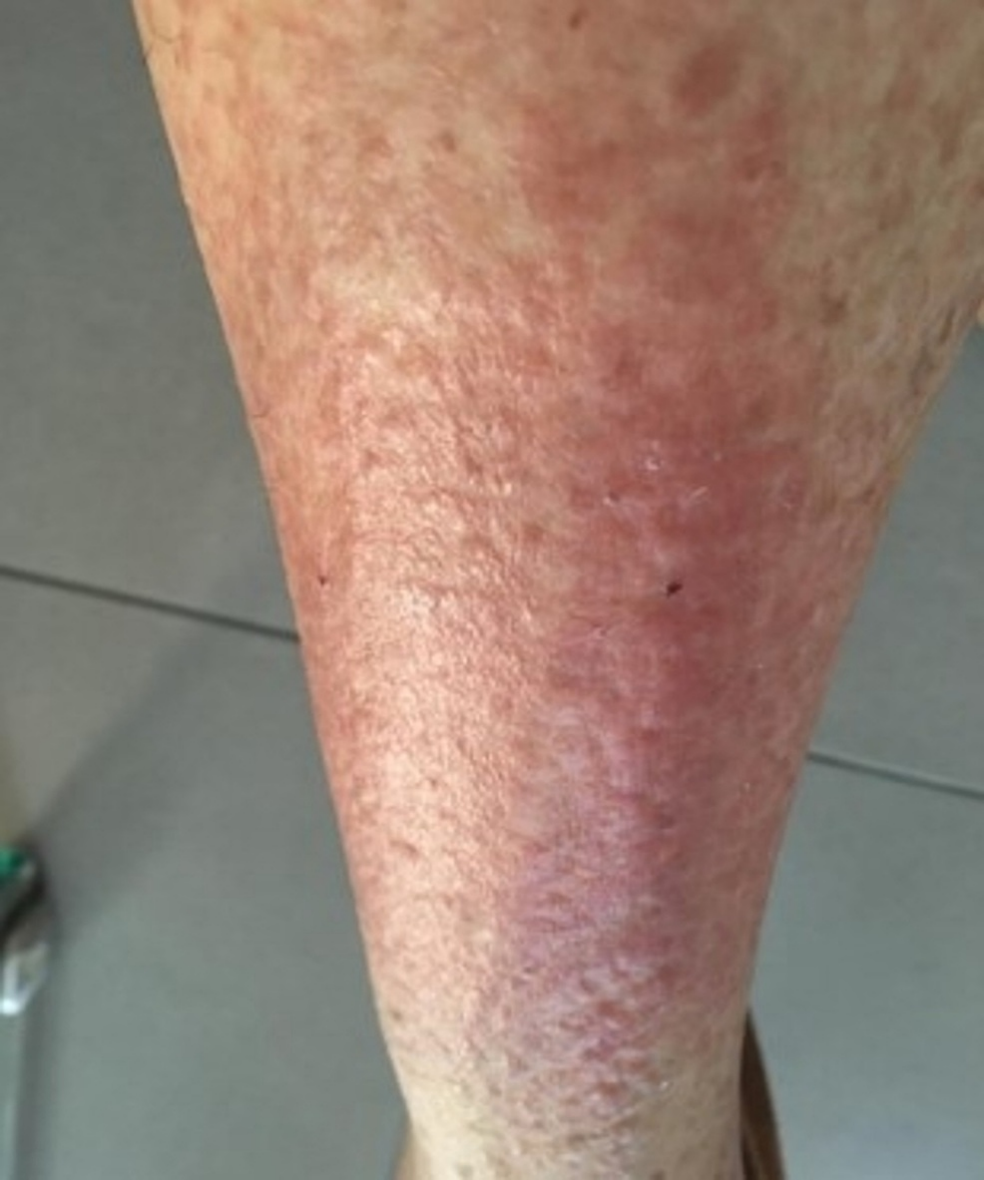
Dermatologic manifestations after empagliflozin initiation

Nearly the same reaction appeared 14 days after starting empagliflozin (Figure 2). The skin biopsy was repeated with results being similar to the previously described one. When empagliflozin was withheld, complete recovery was achieved in three weeks.

The patient was ultimately switched to liraglutide; however, she did not tolerate the medication due to gastrointestinal side effects (nausea and vomiting). The medication was stopped in less than two weeks.

The patient was managed with a careful low carbohydrate diet in between the termination and initiation of the alternative oral anti-diabetic agents. She kept a relative follow-up on home blood glucose readings, but she eventually always needed anti-diabetic drugs for better control. By the time the options of oral agents were exhausted, the patient required basal insulin injections on certain evenings based on the sugar level.

Finally, the patient was switched to insulin glargine 100 units/mL and pre-prandial aspart insulin that she tolerated very well. Upon the follow-up visit to the clinic after three weeks, home blood glucose was well regulated, without any skin manifestations.

## Discussion

FDE is a distinctive type of cutaneous drug reaction that would recur at the identical location upon re-exposure to the same offending drug. It is considered to be a type-IV (delayed) hypersensitivity reaction and is mediated through agent-induced sensitization followed by the development of sensitized cytotoxic T-cells. Most of the cases occur in the second to fourth decades of life [3].

FDE can occur at various sites of the body with a predilection for the extremities, especially proximal regions [4]. FDE across the lower back, sacral area, hips, and mucosal surfaces, like the oral and genital regions, have also been reported. Therefore, skin lesions occur at the same site each time the drug is administered. Prior studies have shown 16.5-30% have involvement of the genital area with a bullous variant in 6.7% [5,6]. Although considered to be infrequent, FDE with bullous formation has been previously shown to encompass the mucus membrane [7]. The rare and severe atypical variants of FDE, including multiple non-pigmented and the previously described generalized bullous forms, share clinical features with Stevens-Johnson syndrome/toxic epidermal necrolysis. Systemic symptoms, such as fever and malaise, are usually absent. Acute lesions mostly appear within eight hours after drug administration but, in certain cases, it might take up to two weeks after the exposure [8]. After discontinuation of the offending drug, lesions settle in seven to 10 days, with a residual gray/brown or slate gray postinflammatory hyperpigmentation. This is due to the overproduction of disordered melanin distribution with respect to the adjacent keratinocytes.

The induction of FDE is dependent on the exogenous agent. It is never caused spontaneously or following a certain type of infection. Several drugs can induce FDE, and the pharmacological agents most frequently associated with FDE include antibiotics (such as trimethoprim-sulfamethoxazole, tetracyclines, penicillins, and quinolones), nonsteroidal anti-inflammatory drugs (NSAIDs), and hypnotics [9]. Rare cases of FDE induced by anti-diabetic agents have also been reported as it was with this case. There has been an argument for the role of genetic predisposition in the manifestation of FDE with human leukocyte antigen (HLA) class I and II exhibiting statistically significant antigenic frequencies [10]. FDE due to sulfamethoxazole has been genetically linked to the HLA-A30-B13-Cw6 haplotype [11]. Genetic testing was not available at our disposal for this patient.

The diagnosis of typical FDE is based upon the lesion’s morphology and history. Skin provocation tests can be performed to identify the culprit drug when the history is unclear, or if multiple medications are suspected [9]. A skin biopsy for histological examination might be indicated in patients with unusual clinical presentations, for example, the atypical FDE forms. The histological findings that suggest the diagnosis is described as lichenoid tissue reaction with pigmentary incontinence related to the accumulation of melanin in the upper dermis and dermal macrophages [12].

There are isolated reports of FDE lesions reactivated by chemically unrelated drugs, a phenomenon known as polysensitivity [13]. This case highlights a polysensitive skin reaction against chemically unrelated drugs, recurring at the same anatomic site. It is very unusual occurrence when a patient develops hypersensitivity to different classes of anti-diabetic medications that characteristically re-emerges on the same site each time a different drug is taken.

Drugs belonging to similar classifications and having similar chemical structures can show such cross-reactivity in patients with FDE. The fact that this patient developed the same cutaneous lesions every time with the use of four different, pharmacologically unrelated, anti-diabetics, raises the hypothesis of a common excipient or film coating ingredient causing this monomorphic rash. Consequently, magnesium stearate was found to be present in all of the four oral anti-diabetic formulations that our patient had used (Table 1). Based on this evidence, it is likely that it was the responsible excipient causing the FDE. The fact that the insulin used does not show any magnesium stearate in its composition might be the reason behind the tolerance towards the therapy. Liraglutide did not show any magnesium stearate content as well; however, the early cessation of the treatment, in less than two weeks, due to the mentioned side effects, halts the interpretation of liraglutide’s impact. It is interesting to note that magnesium stearate is one of the ingredients in trimethoprim-sulfamethoxazole, tetracyclines, quinolones, penicillin, and ibuprofen (NSAID); all of which have been previously described in FDE as previously indicated [9].

**Table 1:** The excipient compositions of the seven antidiabetic agents that the patient received

Anti-diabetics	Metformin	Gliclazide	Vildagliptin	Empagliflozin	Liraglutide	Glargine	Aspart
Compositions	-Magnesium stearate -Povidone K 30 -Hypromellose	-Magnesium stearate -Calcium hydrogen phosphate dihydrate -Maltodextrin -Hypromellose -Anhydrous colloidal silica	-Magnesium stearate -Lactose anhydrous -Cellulose microcrystalline -Sodium starch glycolate (type A)	-Magnesium stearate -Lactose monohydrate -Microcrystalline cellulose -Hydroxypropylcellulose -Croscarmellose sodium -Colloidal anhydrous silica	-Disodium phosphate dihydrate -Propylene glycol -Phenol -Water for injections	-Zinc chloride -Metacresol -Glycerol -Hydrochloric acid -Sodium hydroxide -Water for injections	-Glycerol -Phenol -Metacresol -Zinc chloride -Disodium phosphate dehydrate -Sodium chloride Hydrochloric acid -Sodium hydroxide Water for injections

To the best of our knowledge, this is the first case report of FDE induced by magnesium stearate. In fact, this chemical is widely used in the pharmaceutical industry for its anti-binder properties. It is used for the preparation of numerous drugs as it diminishes their adherent ability and improves the gradual absorption of the active form and its sustained release. Some cases had been published reporting allergic skin reaction secondary to the use of this substance, but not FDE [14]. A previous case described polysensitivity involving a patient with fixed doses of vildagliptin - metformin and ofloxacin - ornidazol [15]. A rash appeared around the mouth and the oral mucosal that subsided after approximately two months from stopping the oral anti-diabetics. A residual dark pigmentation was left in its place. As a result, it was hypothesized, through a study by Bettini et al, that the cumulative consumption of titanium dioxide nanoparticles (TiO2NPs), found in the mentioned drugs and some foods, could be the reason behind this FDE [16]. This is similar to our case, especially with the same two anti-diabetics used by our patient; these drugs are of different pharmacological families. Ofloxacin and ornidazol are also of different drug classes, which highlights the possibility of having an excipient as a causative agent.

Another case report that exemplifies polysensitivity was described in a young lady that developed FDE after taking her first dose of metronidazole [17]. An identical eruption after taking doxycycline had already appeared a year earlier as well. Biopsy results from the erythematous outer zone revealed lichenoid dermatitis as seen in our case.

Likewise, diffused macular erythematous lesions were reported in a middle-aged woman one week after starting sitagliptin [18]. The cessation of the drug lead to the resolution of the erythema and Dipeptidyl Peptidase-4 Inhibitor (DPP4i) was considered the likely suspect when an oral provocation test resulted in a reactivation of the lesions through the previously sensitized CD8+ T-cells. A provocation test was not done in our patient based on the patient’s preferences initially and having two confirmatory skin biopsies.

Additionally, FDE caused by dapagliflozin use has been reported. Withdrawing the offending agent, after nearly one year of intake, resulted in a resolution of forearm erythematous lesions in a patient who was known to have psoriatic disease [19]. Provocation test with the same anti-diabetic drug lead to the re-emergence of the lesions. This raises the possibility of pathological skin conditions predisposing to the development of FDE. In our case, empagliflozin was the sodium/glucose cotransporter-2 inhibitor (SGLT2i) drug used. However, our patient did not have any previous chronic dermatologic conditions.

Another case reported FDE with the use of metformin extended-release; the patient developed rounded red lesions on the palms and soles that ceased after discontinuation of the drug [20]. However, although metformin was high on the list as the causative agent, the patient, in this case, was on several other non-diabetic drugs as well. In our case, the patient was only on a single anti-diabetic drug each time she developed the skin manifestations.

The treatment of FDE is mainly symptomatic and directed towards the relief of pruritus. The most important aspects of the management are prompt diagnoses and identification of the culprit drug. After drug discontinuation, most lesions resolve in a couple of weeks without the need for medical intervention. Nevertheless, in severely symptomatic patients, medical treatment may be required. This includes systemic antihistamines, topical or systemic corticosteroids, depending on the disease gravity and extension. The residual post-inflammatory hyperpigmentation, as a result of the reactive hypermelanosis of the skin that occurs secondary to the cutaneous inflammation, tends to resolve slowly over time. Photoprotection is important to avoid any exacerbation of the FDE.

## Conclusions

Having polysensitivity reactions among anti-diabetic medications should not exclude FDE as a diagnosis. This reaction can occur in chemically unrelated drugs likely secondary to an inactive ingredient. This is the first time where magnesium stearate is suspected as a cause of FDE. Its widespread use has led us to report this case to highlight the importance of investigating excipient as a trigger for FDE. The most important aspects of the disease management are prompt diagnoses and discontinuation of the culprit drug. It is also important to avoid all possible chemically related agents and cross-reactive drugs.
